# Tra1 controls the transcriptional landscape of the aging cell

**DOI:** 10.1093/g3journal/jkac287

**Published:** 2022-10-31

**Authors:** Khaleda Afrin Bari, Matthew D Berg, Julie Genereaux, Christopher J Brandl, Patrick Lajoie

**Affiliations:** Department of Anatomy and Cell Biology, The University of Western Ontario, London, ON N6A 5C1, Canada; Department of Biochemistry, The University of Western Ontario, London, ON N6A 5C1, Canada; Department of Anatomy and Cell Biology, The University of Western Ontario, London, ON N6A 5C1, Canada; Department of Biochemistry, The University of Western Ontario, London, ON N6A 5C1, Canada; Department of Biochemistry, The University of Western Ontario, London, ON N6A 5C1, Canada; Department of Anatomy and Cell Biology, The University of Western Ontario, London, ON N6A 5C1, Canada

**Keywords:** chronological aging, yeast, SAGA complex, Tra1, peroxisomes

## Abstract

Gene expression undergoes considerable changes during the aging process. The mechanisms regulating the transcriptional response to cellular aging remain poorly understood. Here, we employ the budding yeast *Saccharomyces cerevisiae* to better understand how organisms adapt their transcriptome to promote longevity. Chronological lifespan assays in yeast measure the survival of nondividing cells at stationary phase over time, providing insights into the aging process of postmitotic cells. Tra1 is an essential component of both the yeast Spt-Ada-Gcn5 acetyltransferase/Spt-Ada-Gcn5 acetyltransferase-like and nucleosome acetyltransferase of H4 complexes, where it recruits these complexes to acetylate histones at targeted promoters. Importantly, Tra1 regulates the transcriptional response to multiple stresses. To evaluate the role of Tra1 in chronological aging, we took advantage of a previously characterized mutant allele that carries mutations in the *TRA1* PI3K domain (*tra1_Q3_*). We found that loss of functions associated with *tra1_Q3_* sensitizes cells to growth media acidification and shortens lifespan. Transcriptional profiling reveals that genes differentially regulated by Tra1 during the aging process are enriched for components of the response to stress. Notably, expression of catalases (*CTA1*, *CTT1*) involved in hydrogen peroxide detoxification decreases in chronologically aged *tra1_Q3_* cells. Consequently, they display increased sensitivity to oxidative stress. *tra1_Q3_* cell*s* are unable to grow on glycerol indicating a defect in mitochondria function. Aged *tra1_Q3_* cell*s* also display reduced expression of peroxisomal genes, exhibit decreased numbers of peroxisomes, and cannot grow on media containing oleate. Thus, Tra1 emerges as an important regulator of longevity in yeast via multiple mechanisms.

## Introduction

*Saccharomyces cerevisiae* has been extensively studied as a eukaryotic model for lifespan regulation since many of the underlying molecular mechanisms are conserved from yeast to mammals (Mortimer and Johnston 1959; Fontana *et al.* 2010; Kaeberlein 2010; Mirisola and Longo 2022). Chronological lifespan (CLS, defined as the extent of time nondividing cells survive in a nutrient-deprived environment; MacLean *et al.* 2001; Laun *et al.* 2006; Murakami *et al.* 2008) and replicative lifespan (the number of cell divisions before replicative senescence; Steinkraus *et al.* 2008) are 2 distinct experimental aging models (Laun *et al.* 2006). CLS experiments are carried out by culturing cells for a substantial period at the stationary phase. During chronological aging, yeast cells go through distinct growth phases: the mid-log phase, diauxic shift, and stationary phase. Each growth phase is illustrated by divergent metabolic activities and gene expression profiles, which are analogous to crucial characteristics of mammalian aging cells including cell cycle arrest, increased respiratory activity, and lipid and protein homeostasis (Tissenbaum and Guarente 2002; Beach *et al.* 2013, 2015; Medkour and Titorenko 2016; Chadwick and Lajoie 2019; Chadwick *et al.* 2020; Mohammad *et al.* 2020). Studies of yeast chronological aging have enabled researchers to identify several key pathways that regulate longevity (Longo and Fabrizio 2012) such as Ras/Pka (Longo 1999) and Sch9/Tor (Fabrizio *et al.* 2001) and the role of sirtuins (Kaeberlein *et al.* 1999), not only in yeast but also in other organisms.

How cells modulate their gene expression in response to stresses including aging involves all components of the transcriptional machinery. The recruitment of RNA polymerase II to promoters is tightly regulated by general transcription factors, gene-specific activators, and coactivators. Coactivators are multisubunit protein complexes that interface between general transcription factors and gene-specific activators (Kingston *et al.* 1996; Eberharter and Becker 2002; Helmlinger *et al.* 2011) and/or regulate modification of histone proteins and nucleosome remodeling (Roberts and Winston 1997; Rando and Winston 2012). Thus, coactivators play a vital role in global gene regulation. Spt-Ada-Gcn5 acetyltransferase (SAGA) and nucleosome acetyltransferase of H4 (NuA4) are prototypical multisubunit coactivator protein complexes that are conserved among eukaryotic organisms. SAGA and NuA4 contain lysine acetyltransferases Gcn5 and Esa1, respectively, which acetylate both histone and nonhistone proteins (Grant *et al.* 1997; Clarke *et al.* 1999; Steunou *et al.* 2014; Downey 2021). Transcriptome analyses for yeast strains lacking the SAGA components Gcn5 or Spt3 revealed that 10% of the stress-related genome is controlled by the SAGA complex (Huisinga and Pugh 2004). SAGA and NuA4 also participate in other non-transcriptional activities, such as telomere maintenance and DNA repair (Bird *et al.* 2002; Downs *et al.* 2004; Lin *et al.* 2008; Atanassov *et al.* 2009; Cheng *et al.* 2018, 2021). In addition, SAGA contains a deubiquitinating module (DUB) that includes the ubiquitin protease Ubp8 that cleaves ubiquitin from histone H2B (Henry *et al.* 2003; Morgan *et al.* 2016) and other targets. The additional targets for deubiquitination by the mammalian homolog of Ubp8, USP22, are particularly noteworthy for their role in cancer progression (Prokakis *et al.* 2021; Ning *et al.* 2022; De Luca *et al.* 2022).

Tra1 is uniquely found in both the SAGA and NuA4 complexes (Saleh *et al.* 1998; Grant *et al.* 1998; Allard *et al.* 1999). Tra1 belongs to the phosphoinositide-3-kinase-related kinase (PIKK) family, which includes ataxia telangiectasia mutated (ATM; Tel1 in *S. cerevisiae*), ataxia telangiectasia, and Rad3 related (ATR; Mec1 in *S. cerevisiae*), the DNA-dependent protein kinase catalytic subunit (DNA-PKc), mammalian target of rapamycin (Tor1 and Tor2 in *S. cerevisiae*), and SMG-1 (suppressor with morphological effect on genitalia family member) (Smith and Jackson 2003; Hill and Lee 2010; Shiloh and Ziv 2013). PIKK proteins have 4 common principal domains: an N-terminal **H**untingtin, elongation factor 3 (**E**F3), protein phosphatase 2A (PP2**A**), and **T**OR1 (HEAT), followed by FRAP-ATM-TRRAP (FAT), phosphatidylinositol 3-kinase (PI3K), and FRAP-ATM-TRRAP C-terminus (FATC) domains (Keith and Schreiber 1995; Bosotti *et al.* 2000; Mordes *et al.* 2008). Approximately, half of the Tra1 molecule consists of helical HEAT repeats and this section interacts with activator proteins to initiate transcription (Brown *et al.* 2001; Bhaumik *et al.* 2004; Knutson and Hahn 2011; Lin *et al.* 2012). A helical FAT domain located C-terminal to the HEAT repeats wraps the N-terminal section of the PI3K domain (Díaz-Santín *et al.* 2017; Sharov *et al.* 2017). At the C-terminus, PIKK family members share a highly conserved phosphoinositide-3-kinase (PI3K) domain. The PI3K domain consists of N- and C-terminal subdomains with a cleft between these 2 lobes, where ATP binds in the catalytically active PIKK family members (Huse and Kuriyan 2002; Taylor and Kornev 2011; Pavletich and Yang 2013). In all PIKK family members except Tra1/TRRAP, the PI3K domain regulates cell signaling by phosphorylating downstream target proteins. The PI3K domain of Tra1 lacks residues essential for ATP binding and phosphate transfer and therefore has no demonstrable kinase activity (Saleh *et al.* 1998; Nelson *et al.* 2006; Mutiu *et al.* 2007). Interestingly, however, mutation of conserved arginine residues to glutamine (termed *tra1_Q3_*) in the kinase cleft domain results in slow growth, increased stress sensitivity, and decreased transcription of SAGA-regulated genes supporting a role for this pseudokinase domain (Berg *et al.* 2018).

In light of the importance of epigenetic changes in aging (Booth and Brunet 2016; Kane and Sinclair 2019), the involvement of SAGA and NuA4 in stress response processes, and the unique role of Tra1 in both SAGA and NuA4 (Helmlinger *et al.* 2011; Cheung and Díaz-Santín 2019), we hypothesized that Tra1 contributes to longevity by regulating the transcriptional responses to stress associated with aging. Indeed, using a loss-of-function mutant, we find that Tra1 significantly alters the transcriptional landscape of the aging cell. Tra1 therefore emerges as a new regulator of the chronological aging process in yeast.

## Materials and methods

### Drugs

Oleic and myristic acid and diamide were purchased from Milipore-Sigma. Propidium iodide was from Thermo Fisher Scientific. 2-(*N*-morpholino)ethanesulfonic acid (MES) buffer solution was from Alfa Aesar.

### Strains and plasmids

Yeast strains were constructed in the W303A background or BY4742 and are described in Supplementary Table 1. Strains expressing either wild-type FLAG-tagged *TRA1* or *tra1_Q3_* were created by integrating an *Sph*I-*Sac*I fragment from pCB2527 or pCB2537, respectively, into W303A and selecting *HIS+* cells expressing either wild-type *URA3*-FLAG-tagged *TRA1* or *tra1_Q3_* PI3K domain and YCplac111-*DED1pr-YHR100* as previously described (Berg *et al.* 2018). yemRFP-SKL was generated by cloning yemRFP-SKL into the *Spe*I/*Sal*I sites of pRS416-GDP (Mumberg *et al.* 1995).

### Culture conditions and cell viability assay

Both the *TRA1* and *tra1_Q3_* yeast cells (W303A derivatives) were grown to saturation overnight in synthetic complete medium (2% glucose with appropriate selection). An aliquot of the overnight cultures was diluted in fresh media and grown at 30°C in a rotating drum. Cell viability assays were conducted using propidium iodide as described previously (Chadwick *et al.* 2016). Briefly, cells were washed and resuspended in phosphate-buffered saline (PBS) containing 1 mM of propidium iodide. The positive control was prepared by boiling cells for 10 min before resuspending cells in propidium iodide staining solution. Unstained cells were used as a negative control. All samples were incubated for 10 min at room temperature in 96-well plates before imaging on a Gel doc system (Bio-Rad). The optical density (OD_600_) was measured using a BioTek Epoch 2 microplate reader. Both the *TRA1* and *tra1_Q3_* cells were cultivated for several days and the cell viability of the chronologically aged cells was quantified at various time points throughout the aging process. Alternatively, the CLS assay was performed using caloric restricted medium (0.1% glucose), medium buffered with 0.1 M MES buffer, or media containing 0.1% oleic acid, 0.1% myristic acid, or 2% glycerol. To assess sensitivity to acetic acid, cells were grown in synthetic complete medium for 4 days and treated with acetic acid for 200 min with concentrations up to 0.08 mM and the cell viability assessed using propidium iodide. Survival rates were computed using the mean gray value from images along with OD_600_ using the ANALYSR program (Chadwick *et al.* 2016).

### Growth assays

A single colony of yeast strain was inoculated and grown overnight in 2% synthetic medium without leucine at 30°C, 220 rpm. Overnight cultures were serially diluted and OD_600_ measured using a spectrophotometer. Cell cultures were normalized to OD_600_ 0.1- and 5-fold serial dilutions were spotted on solid media. Plates were incubated for 2 days at 30°C before taking images using a colony imager (S&P Robotics). Growth was quantified as previously described (Petropavlovskiy *et al.* 2020).

### RNA isolation and sequencing

Both *TRA1* and *tra1_Q3_* cells were grown to saturation overnight, diluted 10-fold in 2% synthetic minus leucine medium and grown for 4 h at 30°C in a rotating drum. An aliquot of the cells was spun down at day 0 and day 3 and stored at −80°C. Total RNA was isolated from cells using the RiboPure yeast kit (Thermo Fisher Scientific) according to the manufacturer’s instructions. RNA samples were prepared with 3 replicates for each genotype and time point. Quality of the RNA samples were analyzed using a Bioanalyzer to ensure a RIN value of 8 or higher.

Total RNA sequencing analysis was conducted by Azenta Life Sciences. RNA from each sample was converted into single stranded Illumina TruSeq cDNA libraries with poly dT enrichment. Libraries were sequenced on an Illumina HiSeq and each sample yielded between 27.5 and 39.7 million 150-bp paired-end sequencing reads. The raw reads and RNA count data were deposited in NCBI’s Gene Expression Omnibus (Edgar *et al.* 2002).

### Quality control, trimming, read alignment, and differential gene expression analysis

Read quality was analyzed using FastQC (www.bioinformatics.babraham.ac.uk/projects/fastqc/). Trimmomatic default settings were used to trim low quality bases and adapter sequences (Bolger *et al.* 2014). Next, reads were aligned with the reference genome of *S. cerevisiae S288C* sequence (R64-2-1; www.yeastgenome.org/) using STAR (Dobin *et al.* 2013). Only uniquely mapping reads were retained. featureCount was used to count the reads mapping to each gene (Liao *et al.* 2014). Differential expression analysis was performed with DESeq2 (Love *et al.* 2014). Differentially expressed genes with a Benjamini–Hochberg adjusted *P*-value cutoff of ≤0.05 were considered for further analysis.

To determine the enrichment of Rlm1 targets, promoter analysis of differentially expressed genes (log_2_ fold change <2 and *P*-value cutoff of ≤0.05) was performed using Yeastract (www.yeastract.com) (Monteiro *et al.* 2020).

### Fluorescence microscopy

*TRA1* and *tra1_Q3_* cells expressing yemRFP-SKL were grown to saturation overnight, diluted 10-fold in 2% synthetic minus leucine medium and grown for 4 h at 30°C in a rotating drum. An aliquot of the cells was then spun down, washed, resuspended in PBS, and transferred to a Nunc Lab-Tek chamber slide. Cells were imaged using a Zeiss Axiovert A1 wide-field fluorescence microscope with 63× 1.4 NA oil objective using a Texas Red filter (586 nm excitation/603 nm emission) and an AxioCam ICm1 R1 CCD camera. ImageJ was used to analyze the images (Schneider *et al.* 2012).

## Results

### Functional Tra1 is required for chronological aging

**Fig. 1.**
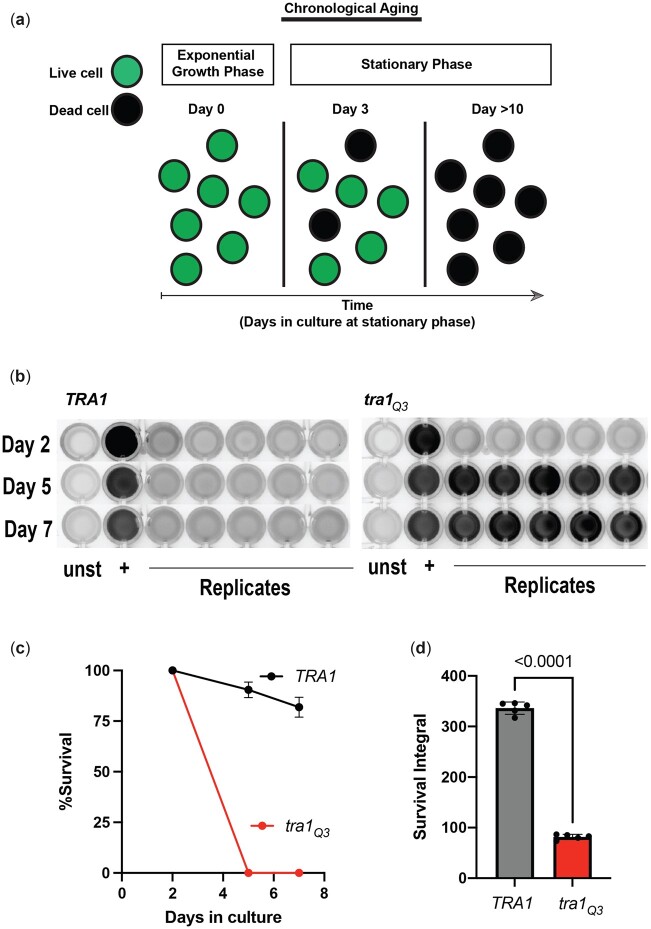
Functional Tra1 is required for chronological aging. a) CLS is defined as the amount of time yeast cells survive at stationary phase. Experimentally, longevity is assessed by labeling cells with a viability dye such as propidium iodide at various time points during the aging process. Adapted from Chadwick *et al.* 2016. b) *tra1_Q3_* cells display reduced CLS. *TRA1* and *tra1_Q3_* cells were grown for the indicated times in standard synthetic complete medium and stained with propidium iodide to measure cell survival. Stained cells were imaged in a 96-well plate. For each time point, a negative control (unstained cells), a positive control (boiled cells), and 5 replicates were analyzed. c) Normalized survival rates over the aging process and d) survival integral are shown in the bar graph. Significance was assessed using an unpaired Student *T*-test.

Tra1 is essential in yeast (Saleh *et al.* 1998) with the exception of the fission yeast *Schizosaccharomyce pombe*, which expresses a second *TRA2* isoform (Helmlinger *et al.* 2011; Elías-Villalobos, Fort, *et al.* 2019). To determine the role of Tra1 in the aging process, we took advantage of a loss-of-function mutant that we previously characterized termed *tra1_Q3_* (Berg *et al.* 2018). *tra1_Q3_* contains 3 glutamine substitutions of arginine residues 3,389, 3,390, and 3,456 proximal or within the putative ATP-binding cleft of the PI3K domain. *tra1_Q3_* results in slow growth, increased stress sensitivity, transcriptional defects and impaired SAGA and NuA4 complex assembly (Berg *et al.* 2018; Razzaq *et al.* 2021). CLS was assessed by labeling cells with the viability dye propidium iodide (PI) at different time intervals during aging (Fig. 1a). *tra1_Q3_* cells display significantly reduced lifespan when compared to their wild-type counterparts (Fig. 1, b–d). Viability of *tra1_Q3_* cells in exponentially growing culture is comparable to wild type. *tra1_Q3_* cells begin to decline in viability when cells reach the stationary phase indicating that a functional *TRA1* allele is required to extend CLS.

**Fig. 2.**
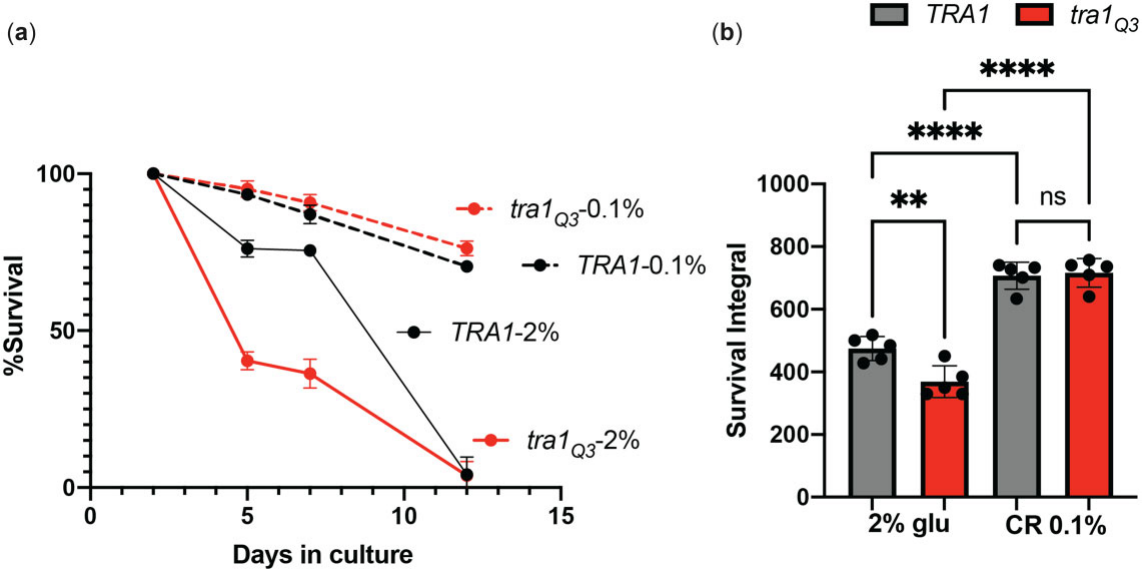
Tra1 function is dispensable for lifespan extension by caloric restriction. a) *TRA1* and *tra1_Q3_* cells were grown for the indicated time in standard synthetic complete medium containing either 2% glucose or 0.1% glucose (CR) and stained with propidium iodide to measure cell survival. Stained cells were imaged in a 96-well plate. For each time point, a negative control (unstained cells), a positive control (boiled cells), and 5 replicates were analyzed. b) Normalized survival rates over the aging process and calculated survival integral are shown in graphs. Significance was assessed using a 1-way ANOVA followed by a Tukey’s multiple comparison test. **P < 0.01 and ****P < 0.0001.

Since caloric restriction efficiently increases longevity in yeast and other organisms (Jiang *et al.* 2000; Fontana *et al.* 2010; Choi *et al.* 2013; Arlia-Ciommo *et al.* 2014; Leonov *et al.* 2017; Campos *et al.* 2018), we then tested whether functional Tra1 is required for lifespan extension by caloric restriction. Caloric restriction is achieved by growing the cells in 0.1% glucose medium whereas complete medium contains 2% glucose. We found that when grown under caloric restriction conditions, *tra1_Q3_* cells display extended lifespan similar to wild-type *TRA1* cells (Fig. 2), suggesting that Tra1 function is dispensable for lifespan extension by caloric restriction.

### *tra1_Q3_* cells are more sensitive to acetic acid

**Fig. 3.**
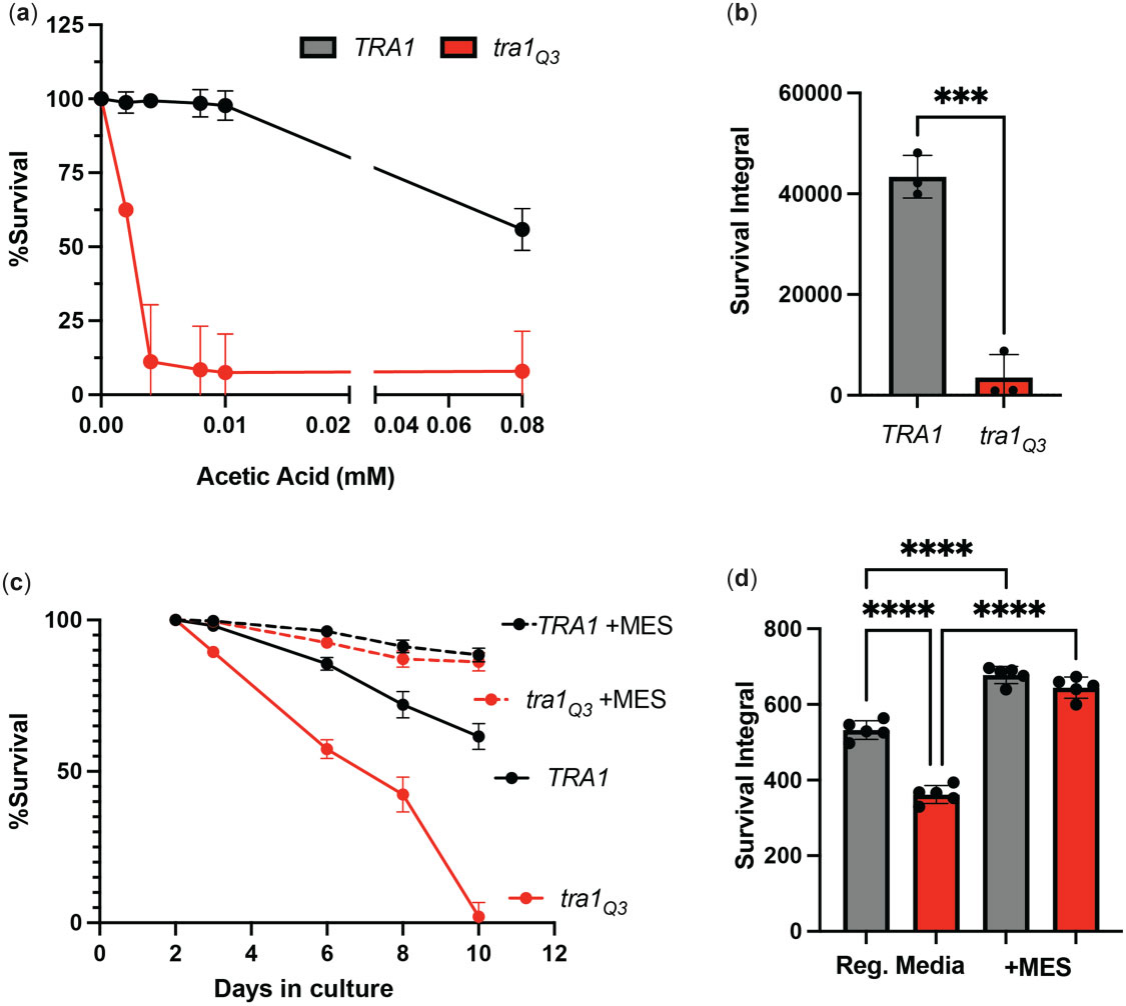
Increased sensitivity of *tra1_Q3_* cells to media acidification is linked to shortened lifespan. a) *tra1_Q3_* cells display increased sensitivity to acetic acid. *TRA1* and *tra1_Q3_* cells were grown for 4 days and subsequently treated with the indicated concentrations of acetic acid for 200 min and stained with propidium iodide to measure cell survival. b) Normalized survival rates upon acetic acid treatment were calculated and survival integrals are shown. c) *TRA1* and *tra1_Q3_* cells were grown for the indicated time in standard synthetic complete medium with or without 0.1 M MES and stained with propidium iodide to measure cell survival. Stained cells were imaged in a 96-well plate. For each time point, a negative control (unstained cells), a positive control (boiled cells) and 5 replicates were analyzed. d) Normalized survival rates over the aging process and calculated survival integrals are shown. Significance was assessed using a 1-way ANOVA followed by a Tukey’s multiple comparison test. ***P < 0.005 and ****P < 0.0001.

Culture media acidification is a predominant cell-extrinsic factor associated with cell death during chronological aging (Burtner *et al.* 2009; Burhans and Weinberger 2009; Hu *et al.* 2014; Deprez *et al.* 2018; Chaves *et al.* 2021). During chronological aging, acetic acid is produced following depletion of glucose from the growth media when ethanol is utilized as the main carbon source. Indeed, long-lived mutants, such as *sch9Δ* and *ade4Δ*, show increased resistance to acetic acid (Burtner *et al.* 2009; Matecic *et al.* 2010). Therefore, we sought to determine if increased sensitivity to acidification of the cell culture media by acetic acid explains, at least in part, the shorter lifespan of *tra1_Q3_* cells. To this end, *TRA1* and *tra1_Q3_* cells were grown to stationary phase for 4 days and treated with various concentrations of acetic acid. Indeed, *tra1_Q3_* increases sensitivity to acetic acid compared with wild-type *TRA1* (Fig. 3, a and b). Media acidification during chronological aging is alleviated by buffering the cultures to pH 6 with citrate phosphate or MES, resulting in increased longevity (Burtner *et al.* 2009; Burhans and Weinberger 2009). We found that buffering the aging media pH with MES significantly extended the lifespan of both *TRA1* and *tra1_Q3_* cells (Fig. 3, c and d), suggesting that the inability to respond to acid stress plays an important role in the reduced longevity phenotype associated with the *tra1_Q3_* mutation.

### Tra1 controls the transcriptional landscape of the aging cell

**Fig. 4.**
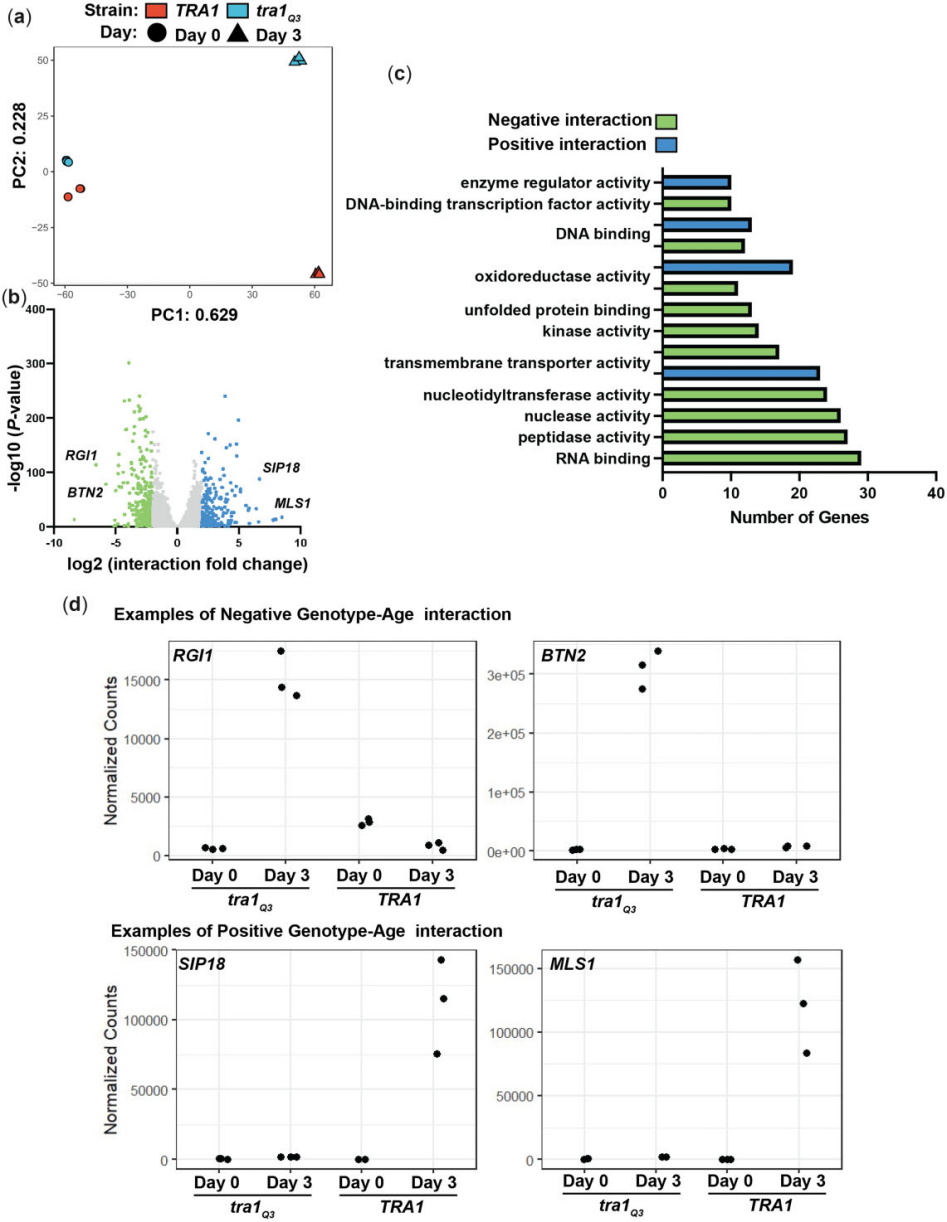
The *tra1_Q3_* mutation alters the transcriptional landscape of aging cells. a) Principal component analysis of centered log ratio of normalized reads from *TRA1* and *tra1_Q3_* cells at day 0 and day 3. Each point represents a single biological replicate (*n* = 3). b) Volcano plot of genes that respond differently to the aging process in the *tra1_Q3_* cells compared to wild-type *TRA1* (coloured for dark points represent *P* < 0.05, log_2_ fold change >1). c) Significantly enriched GO biological processes were determined for genes with both positive and negative age–genotype interaction with log_2_ interaction score >2 (*P* < 0.05) in *tra1_Q3_* cells compared to wild type. d) Examples of genes with positive (*SIP18*, *MLS1*) and negative (*RGL1*, *BTN2*) age–genotype interaction. Normalized RNA sequencing read counts are shown for *TRA1* and *tra1_Q3_* cells at day 0 and day 3.

We previously found that Tra1 regulates the expression of genes associated with several stress responses including cell wall stress and the response to misfolded proteins (Berg *et al.* 2018; Jiang *et al.* 2019; Razzaq *et al.* 2021). Based on findings that *tra1_Q3_* cells have a shorter lifespan, we next sought to determine how the loss of Tra1 function impacts the transcriptional landscape of the aging cell. We performed a global transcriptome analysis using RNA sequencing for TRA*1* and *tra1_Q3_* cells before chronological aging (day 0) and after 3 days at stationary phase (day 3) to identify genes subjected to different transcriptional regulation. Day 3 was selected because the cells have reached stationary phase and still show modest minimal cell death (Supplementary Fig. 1). We found that ∼60% of the variance in the transcriptome data is linked to the aging process and ∼20% is linked to the *tra1_Q3_* mutation (Fig. 4a). The larger distance between *TRA1* and *tra1_Q3_* cells after aging suggests that transcriptional differences between the 2 strains is greater in aged cells. At day 0, 394 genes were statistically differentially regulated (175 downregulated and 219 upregulated) when comparing *TRA1* and *tra1_Q3_* cells (*P* < 0.05, log_2_ fold change >2) (Supplementary Table 2). At day 3, there were 2,026 statistically differentially expressed genes (978 downregulated and 1,048 upregulated) (Supplementary Table 2). When comparing the age–genotype interaction scores (Smith and Kruglyak 2008; Sardi and Gasch 2018), we found 287 genes (161 negative and 126 positive) with a log_2_ score >2.5 and *P* < 0.05, suggesting that these genes differentially respond to the aging process (Fig. 4b) in the 2 genotypes (*TRA1* and *tra1_Q3_*). Genotype (*TRA1* vs *tra1Q3*) by environment (day 0 vs day 3) interaction scores allow us to decipher how different genotypes specifically modulate gene expression during the aging process. Gene Ontology (GO) analysis revealed significant enrichment in several biological processes, including RNA binding, transmembrane transporter activity, oxidoreductase activity, and unfolded protein binding (Fig. 4c). Genes associated with misfolded protein stress (*SSA1*, *HSP26*, *HSP42*, *SSA4*, *MID1*, *KAR2*, *SSA2*, *CPR6*, *HSP60*, *HSC82*, *APJ1*, *HSP10*, *HSP82*) are upregulated in aged *tra1_Q3_* cells compared to wild-type suggesting that these cells exhibit increased proteotoxic stress. Genes with a negative age–genotype interaction score were also enriched in Rlm1 target genes, consistent with our previous results showing that Tra1 controls cell wall integrity (Supplementary Fig. 2) (Berg *et al.* 2018; Razzaq *et al.* 2021). Genes with the highest positive and negative interaction scores are presented in Fig. 4d. Genes upregulated in aged *tra1_Q3_* cells compared to wild type include *RGI1* (involved in metabolism under respiratory conditions) and *BTN2* (a v-snare binding protein). Interestingly, *BTN2* expression is increased under severe ethanol stress (Yamauchi and Izawa 2016), reinforcing the idea that *tra1_Q3_* cells are subject to increased stress during the aging process. Genes with positive age–genotype interaction are upregulated in wild-type *TRA1* compared to *tra1_Q3_* in aged cells. Examples of such genes are *SIP18* and *MLS1*. *SIP18* encodes a phospholipid binding hydrophilin involved in vacuolar membrane fusion and is upregulated during replicative lifespan (Ghavidel *et al.* 2018). *MLS1* encodes a malate synthase involved in the utilization of non-fermentable carbon sources and is also upregulated in replicatively aged cells (Lesur and Campbell 2004).

**Fig. 5.**
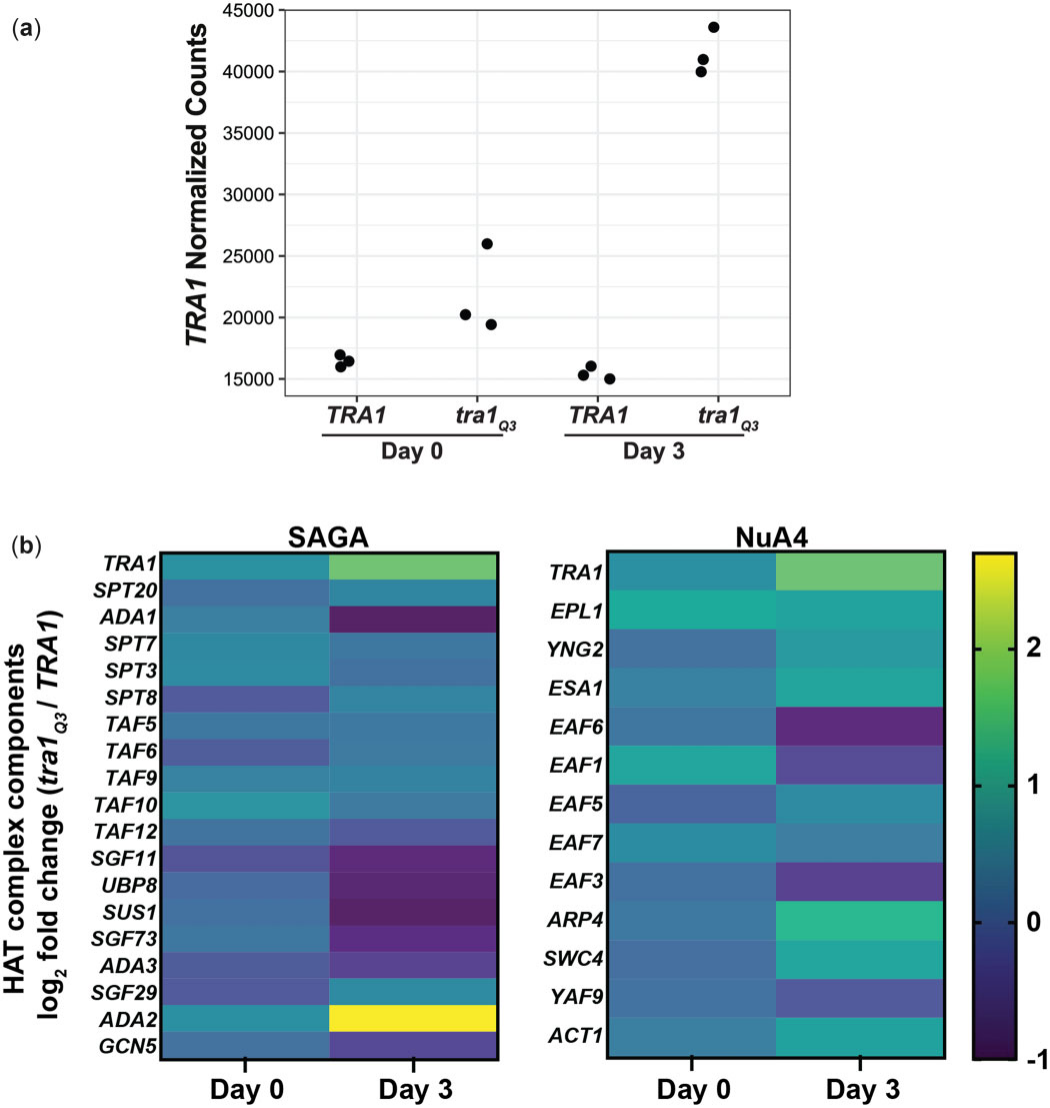
Aged *tra1_Q3_* cells display increased *TRA1* mRNA abundance. a) Normalized RNA sequencing read counts are shown for *TRA1* mRNA in wild-type *TRA1* and *tra1_Q3_* cells at day 0 and day 3. b) Log_2_ fold change (*tra1_Q3_/TRA1*) for the mRNA of genes encoding SAGA and NuA4 components at day 0 and day 3.

We previously showed that cells expressing *tra1_Q3_* increase expression of *TRA1* as a possible compensatory mechanism for the loss of protein function (Berg *et al.* 2018). Here, we observed this phenomenon as *tra1_Q3_* mRNA increased ∼1.7-fold compared to wild-type at day 0. This difference was exacerbated in aged cells as *TRA1* mRNA increased ∼3.2-fold in tra*1_Q3_* cells after aging (Fig. 5a). We also analyzed the mRNA levels of other SAGA and NuA4 components (Fig. 5b). Most striking are the upregulation of *ADA2* in aged *tra1_Q3_* cells and downregulation of components of the SAGA DUB module (*UBP8*, *SUS1*, *SGF73*, *SGF11*). Among the NuA4 components, *ARP4* was upregulated in aged *tra1_Q3_* cells. Interestingly, mutation of *ARP4* affects CLS in combination with deletion of the linker histone Hho1 (Vasileva *et al.* 2021).

**Fig. 6.**
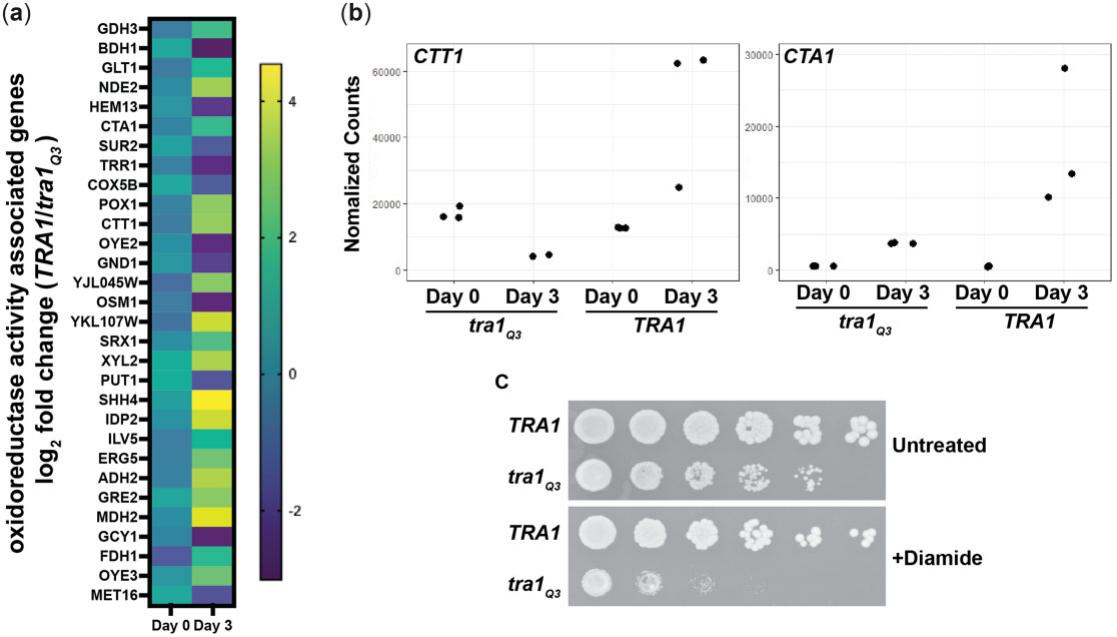
*tra1_Q3_* cells are sensitive to oxidative stress. a) Log_2_ fold change (*TRA1/tra1_Q3_*) for mRNA of genes associated with oxidoreductase activity in wild-type *TRA1* and *tra1_Q3_* cells at day 0 and day 3. b) Normalized RNA sequencing read counts are shown for *CTT1* and *CTA1* in *TRA1* and *tra1_Q3_* cells at day 0 and day 3. c) *tra1_Q3_* cells are sensitive to diamide. *TRA1* and *tra1_Q3_* cells were spotted onto agar plates without (untreated) or with 1 mM diamide.

GO analysis of genes that respond differentially to the aging processing in *tra1_Q3_* cells revealed an enrichment in genes associated with oxidoreductase activity (Fig. 6a). Among those genes were *CTT1* and *CTA1*, which encode 2 versions of catalase in *S. cerevisiae*. Ctt1 is cytoplasmic (Seah and Kaplan 1973) while Cta1 localizes to both the mitochondria and peroxisomes (Petrova *et al.* 2004). Catalase activity is crucial for hydrogen peroxide detoxification and is an important regulator of oxidative stress resistance associated with various conditions, including aging in yeast (Petriv and Rachubinski 2004; Agarwal *et al.* 2005; Mesquita *et al.* 2010; Rona *et al.* 2015; Guaragnella *et al.* 2019). Both genes are substantially upregulated in aged wild-type *TRA1* cells but not in *tra1_Q3_* cells (Fig. 6b). Consequently, *tra1_Q3_* cells were more sensitive to oxidative stress induced by diamide (Fig. 6c). Thus, these data indicate that *tra1_Q3_* cells have reduced capacity to cope with oxidative damage that is usually associated with the aging process (Pan 2011).

**Fig. 7.**
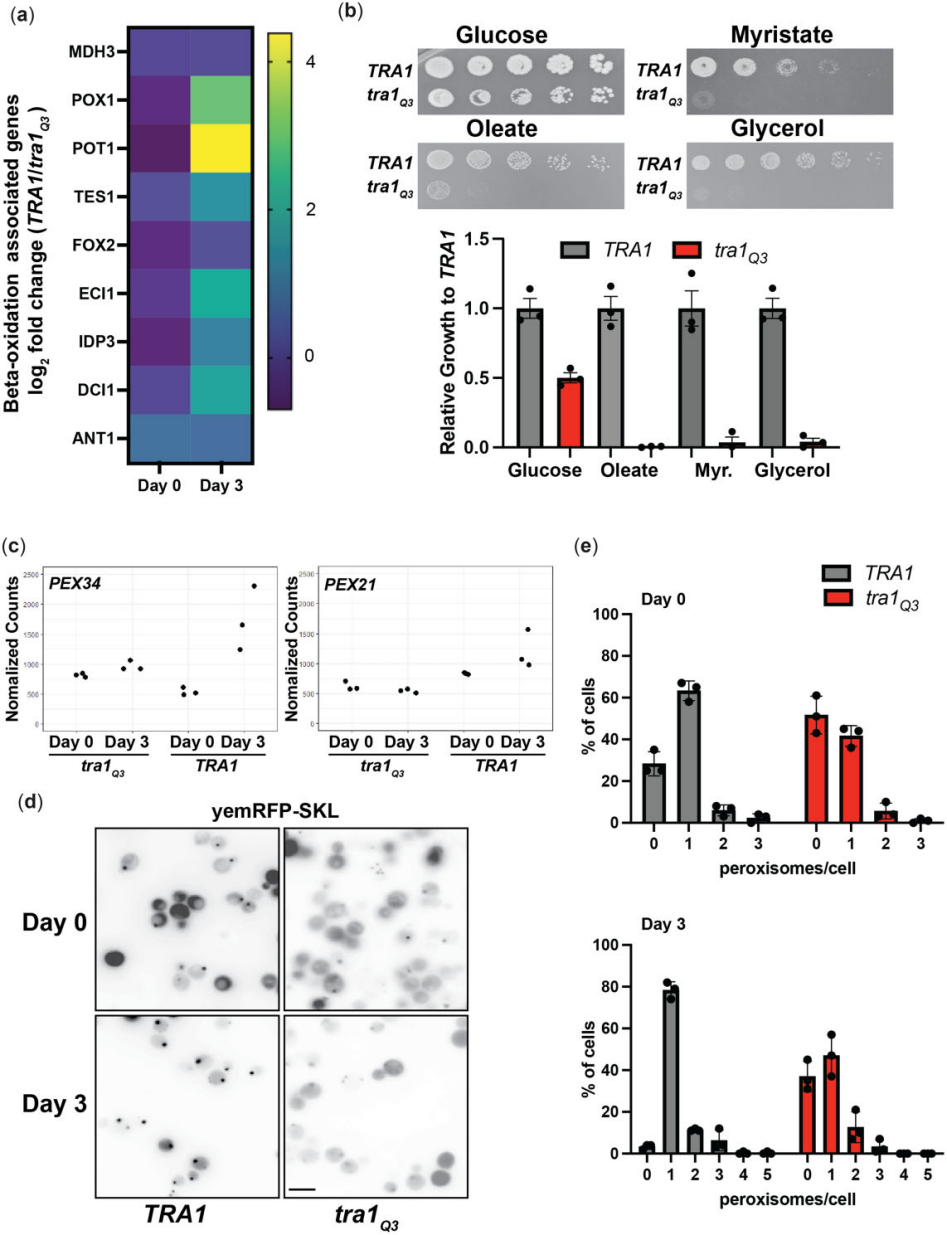
*tra1_Q3_* results in defective peroxisomes. a) Log_2_ fold change (*TRA1/tra1_Q3_*) for beta-oxidation genes at day 0 and day 3. b) *tra1_Q3_* cells show decreased growth in the presence of oleic acid. *TRA1* and *tra1_Q3_* cells were spotted on agar plates containing either glucose (YPD), oleate, myristate, or glycerol as the carbon source. Growth of *tra1_Q3_* cells relative to *TRA1* cells was quantified and is shown in the bar graph. c) Normalized RNA sequencing read counts are shown for *PEX34* and *PEX21* in wild-type *TRA1* and *tra1_Q3_* cells at day 0 and day 3. d) *TRA1* and *tra1_Q3_* cells expressing yemRFP-SKL were imaged using fluorescence microscopy at day 0 and day 3 of the aging process. The number of peroxisomes/cell is shown in bar graphs. Bar: 10 µm.

Several differentially expressed genes were also linked to peroxisomal β-oxidation and fatty acid metabolism (*POT1*, *POX1*, *MLS1*, *DCI1*, *ECI1*, *TES1*) (Fig. 7a). The β-oxidation pathway and peroxisome proliferation are crucial for chronological aging (Lefevre *et al.* 2013). In yeast, β-oxidation solely occurs in peroxisomes (Hiltunen *et al.* 2003) and strains with defective peroxisomes fail to grow in presence of oleate due to their incapacity to use fatty acid as a carbon source (Lockshon *et al.* 2007). We found that *tra1_Q3_* cells were unable to grow on media containing oleate or myristate, suggesting that they are defective in β-oxidation (Fig. 7b). Because acetyl-CoA produced by β-oxidation serves as an energy source only in respiratory-competent strains, we assessed growth on glycerol. We found that tra*1_Q3_* cells are unable to grow on plates containing glycerol (Fig. 7b). This phenotype along with the reduced lifespan was also recapitulated in a different strain background (Supplementary Fig. 3). Since β-oxidation in yeast is performed solely in peroxisomes, we also analyzed the expression of *PEX34* and *PEX21* in *TRA1* and *tra1_Q3_* cells during the aging process. Pex34 regulates peroxisome biogenesis (Tower *et al.* 2011). Pex21 regulates import of protein into the peroxisomal matrix (Purdue *et al.* 1998). Aged *tra1_Q3_* cells show reduced expression of *PEX34* and *PEX21* compared to aged wild-type cells (Fig. 7c). *tra1_Q3_* cells consequently display a reduced number of peroxisomes when labeled with the fluorescent reporter yemRFP-SKL at both day 0 and day 3 (Fig. 7, d and e). Therefore, our data suggest that *tra1_Q3_* cells have defective peroxisome function that might contribute to their reduced CLS.

## Discussion

### A role for Tra1 in chronological aging

Tra1 has been proposed to act as protein–protein hub where it interacts with transcription activators to recruit SAGA and NuA4 coactivator complexes to specific promoters (Elías-Villalobos, Fort, *et al.* 2019). In both SAGA and NuA4, Tra1 regulates multiple stress responses associated with the aging process such as cell wall integrity, ethanol sensitivity, protein misfolding, lipid synthesis, and the response to DNA damage. Thus, Tra1 regulates various aspects of the transcriptional response to the aging process. Interestingly, the Tra1 homolog in *Drosophila*, Nipped-A, is required to maintain proliferative capacity of intestinal stem cells through aging, indicating that Tra1 may play a role in aging across species (Tauc *et al.* 2017).

Caloric restriction impacts multiple stress response pathways that are associated with the aging process, such as those involved in proteotoxic stress (Choi *et al.* 2013; Arlia-Ciommo *et al.* 2018). Here, we also show that caloric restriction alleviates the accelerated aging phenotype associated with the *tra1_Q3_* mutantion. Since our data indicate that the *tra1_Q3_* mutant is subjected to increased stress during aging, it is reasonable to postulate that caloric restriction acts at least in part by alleviating these stresses. Interestingly, while the *tra1_Q3_* cells do not grow on glycerol, their respiratory defect appears to be less than the threshold required for lifespan extension by caloric restriction (Ocampo *et al.* 2012).

This study and previous work using the *tra1_Q3_* mutant (Berg *et al.* 2018; Razzaq *et al.* 2021) support an important regulatory role for the Tra1 PI3K domain, despite the absence of key kinase motifs (McMahon *et al.* 1998; Helmlinger *et al.* 2011). Our previous work showed that Tra1_Q3_ has decreased association with SAGA and NuA components (Berg *et al.* 2018). This included Spt20, which is the preferential Tra1 interactor in both fission and budding yeast (Liu *et al.* 2019; Elías-Villalobos, Toullec, *et al.* 2019; Wang *et al.* 2020). Moreover, Tra1 regulates the incorporation of DUB module components into SAGA (Elías-Villalobos, Toullec, *et al.* 2019). Interestingly, we found decreased expression of DUB components in aged *tra1_Q3_* cells. Whether this is a response to mis-assembly of the complex remains to be determined. Similarly, Leo *et al.* (2018) demonstrated that SAGA, through Gcn5, is essential to properly maintain Ubp8 levels under respiratory conditions. Aging also exacerbated the increase in *TRA1* expression that we previously observed in *tra1_Q3_* cells (Berg *et al.* 2018). The nature of this feedback mechanism remains unclear. Upregulation of *TRA1* is not observed in response to deleting other components of SAGA and NuA4 (Berg *et al.* 2018). *TRA1* expression is also upregulated upon protein misfolding stress (Jiang *et al.* 2019). Therefore, it is reasonable to postulate that increased proteotoxic stress associated with the aging *tra1_Q3_* allele plays a role in this phenotype.

### HAT complexes and lifespan regulation

While we show here that compromising Tra1 function shortens CLS, the role of the different components of the SAGA and NuA4 in aging is complex. Deleting *GCN5* and *SPT20* shortens CLS in winemaking yeast (Orozco *et al.* 2012; Picazo *et al.* 2015). Conversely, deleting the SAGA component *SGF11* extends CLS (Garay *et al.* 2014) indicating that members of HAT complexes can differentially impact the aging process. The SAGA DUB module also regulates replicative aging via its interaction with Sir2 (McCormick *et al.* 2014; Mason *et al.* 2017). Deletion of *GCN5* and its pharmacological inhibition extends replicative lifespan (Huang *et al.* 2020). Therefore, different components of SAGA can differentially affect aging. Arp4 (actin-related protein 4), an essential component of the NuA4 complex, is necessary for chronological aging through its interaction with Hho1 (Miloshev *et al.* 2019). Thus, different components of SAGA and NuA4 can differentially affect aging.

Further studies will be required to determine the role of Tra1 and the impact of the *tra1_Q3_* mutation on the global acetylation of both histone and non-histone substrates. Interestingly, *tra1_Q3_* prevents upregulation of *ACS1* during aging (Supplementary Table 2). *ACS1* encodes a nucleocytoplasmic acetyl-CoA synthetase whose expression increases during chronological aging (Lesur and Campbell 2004; Wierman *et al.* 2017), presumably to maintain the pool of acetyl-CoA required for histone acetylation (Takahashi *et al.* 2006). *ACS1* null cells consequently display reduced CLS (Marek and Korona 2013). This is also in agreement with previous observations that cells deleted for NuA4 and SAGA components (*eaf7Δ* and *gcn5Δ*) display decreased levels of acetyl-CoA (Rollins *et al.* 2017).

Our data also showed that *tra1_Q3_* cells have increased sensitivity to acetic acid (Fig. 3). This is consistent with studies showing the importance of transcriptional regulation in acetic acid tolerance (Dong *et al.* 2017; Vall-Llaura *et al.* 2019; Chaves *et al.* 2021). A direct link to SAGA and NuA4 was suggested by Dong *et al.* (2017) who revealed that acetic acid treatment leads to differential expression of multiple SAGA/NuA4 components. Similar to what we observed, increased levels of *ADA2* and *EPL1* increase sensitivity to acetic acid (Dong *et al.* 2017). Nonetheless, the impact of acid stress in histone acetylation is complex and involves multiple pathways. Vall-Llaura *et al.* (2019) found that deacetylation by the Sir2 histone deacetylase regulates acetic acid tolerance. In addition, defects in the Set3 histone deacetylase complex are associated with increased tolerance to acid stress, suggesting that histone acetylation plays a protective role (Sousa *et al.* 2013). Of note, the most upregulated genes in aged *tra1_Q3_* cells, *BTN2* and *RGI1*, are strongly upregulated by acetic acid (Dong *et al.* 2017). This is consistent with these cells experiencing high acid stress and potentially provides an explanation as to why buffering the growth media’s pH alleviates the aging phenotype associated with the *tra1_Q3_* mutation.

### Tra1, mitochondria, peroxisomes, and the aging process

Here, we showed that several genes important for β-oxidation are decreased in *tra1_Q3_* cells after chronological aging. During chronological aging, yeast cells utilize internal fat stores (Goldberg *et al.* 2009). Ultimately, free fatty acids are the substrate for peroxisomal β-oxidation. This process allows cells to produce acetyl-CoA that is used to generate the ATP in the mitochondria required for survival at stationary phase. Thus, cells that lack mitochondrial respiration (i.e. incapable of growing on glycerol) cannot grow on oleate (Lockshon *et al.* 2007). In our case, the absence of growth of the *tra1_Q3_* strain on medium containing oleate as the carbon source (Fig. 7b) could be linked to deficient mitochondrial respiration. In *S. cerevisiae*, Gcn5 and Ubp8 are also required for respiration (Canzonetta *et al.* 2016; Leo *et al.* 2018), a process essential for chronological aging (Pan *et al.* 2011; Ocampo *et al.* 2012).

We previously found that a *TRA1* mutant displays negative genetic interactions with genes associated with mitochondrial function (Hoke *et al.* 2008). This could reflect altered regulation of the retrograde response associated with aging (Kim *et al.* 2004; Jazwinski 2005; Friis *et al.* 2014; Pogoda *et al.* 2021). The mitochondrial retrograde pathway signals to the nucleus via the Rtg proteins (Rtg1, 2, and 3) to upregulate genes associated with mitochondrial stress (Liao and Butow 1993; Ždralević *et al.* 2015). The canonical retrograde target is *CIT2*, which encodes the peroxisomal isoform of citrate synthase. The retrograde pathway regulates expression of several other peroxisomal proteins (Chelstowska and Butow 1995). Retrograde signaling also upregulates other genes involved in the TCA cycle, such as mitochondrial citrate synthase (*CIT1*), aconitase (*ACO1*), and NAD+-dependent isocitrate dehydrogenase (*IDH1*/*2*). Interestingly, we found that these targets of the retrograde pathway are downregulated in aged *tra1_Q3_* (Supplementary Table 2). While there is debate concerning the incorporation of Rtg2 in the SAGA-like complex (Pray-Grant *et al.* 2002; Adamus *et al.* 2021), our data suggest a role for Tra1 in the regulation of retrograde signaling that could contribute to the changes in peroxisomal gene expression observed in the aged *tra1_Q3_* cells.

Cells carrying deletions in genes encoding other SAGA components display reduced levels of peroxisomal genes (Ratnakumar and Young 2010) suggesting a further link between HAT complexes and peroxisomal biogenesis/functions. Interestingly, cells incompetent for β-oxidation have a less severe aging phenotype than cells devoid of peroxisomes, indicating that other peroxisomal functions are important in regulating CLS (Lefevre *et al.* 2013). Peroxisome proliferation is also associated with the early stage of replicative aging (Deb *et al.* 2022). Free oxidative radicals have long been suggested to regulate the aging process (Harman 1972). Peroxisomes contain catalase that metabolizes hydrogen peroxide and maintains the cellular redox balance (Lismont *et al.* 2015). Efficient import of catalase into the peroxisomes improves longevity in human cells (Koepke *et al.* 2007). Inhibiting human peroxisomal catalase triggers increased mitochondrial reactive oxygen species (Walton and Pizzitelli 2012). In contrast, deletion and pharmacological inactivation of either form of the yeast catalase (*CTT1*, cytosolic; *CTA*1, peroxisomal), is associated with extended CLS (Mesquita *et al.* 2010). It was proposed that lack of catalase in young cells triggers a sublethal level of oxidative stress that allows hormetic adaptation to oxidative stress and, consequently, lifespan extension (Mesquita *et al.* 2010). However, overexpressing catalase also extends the CLS of cells lacking superoxide dismutase (*Δsod1*) showing that catalase levels are indeed important to alleviate oxidative damage associated with aging (Rona *et al.* 2015). Therefore, inability of *tra1_Q3_* cells to properly regulate catalase expression likely has an important role in chronological aging. This is supported by previous studies showing that SAGA plays a major role in the transcriptional response to oxidative stress (Huisinga and Pugh 2004; Sansó *et al.* 2011; Kim *et al.* 2019).

### Perspectives and conclusions

In conclusion, our work offers insight into how Tra1 regulates gene expression associated with several stress pathways such as regulation of cell wall integrity, mitochondria respiration and peroxisomal function that are crucial for chronological aging. Given the growing evidence that chronologically aged yeast cells form a heterogeneous population of quiescent and nonquiescent cells (Allen *et al.* 2006; Aragon *et al.* 2008; Miles *et al.* 2013; Mohammad *et al.* 2020), it will be important to define how transcriptional regulators like Tra1 and other SAGA/NuA4 components regulate gene expression, histone acetylation and chromatin accessibility within the aging population.

## Supplementary Material

jkac287_Supplementary_Data

## Data Availability

Strains and plasmids are available upon request. Gene expression data are available at GEO with the accession number: GSE206033. Supplemental material is available at G3 online.
