# Supplementary material for: Tra1 controls the transcriptional landscape of the aging cell
Source: G3 (Bethesda). 2022 Oct 31;13(1):jkac287. doi: 10.1093/g3journal/jkac287 (PMC9836359; doi:10.1093/g3journal/jkac287)
Supplement: jkac287_Supplementary_Data [file jkac287_supplementary_data.zip › Suppl/Supplemental_Figure_Legends_G3-2022-403832.docx]

**Figure S1: Cell viability of wild-type and *tra1_Q3_* cells at day 0 and 3**. **(A)** *TRA1* and *tra1_Q3_* cells were grown for the indicated time in standard synthetic complete medium and spotted on agar plates. **(B)** *TRA1* and *tra1_Q3_* cells were grown for the indicated times in standard synthetic complete medium and stained with propidium iodide to measure cell survival. Normalized survival rates over the aging process and calculated survival integrals are shown in bar graph.

**Figure S2: Rlm1 associations with differentially downregulated genes in the aged *tra1_Q3_* strain**. The experimental evidence underlying each regulatory association (solid lines for DNA-binding evidence; dashed lines for expression evidence), as well as the sign of the interaction—positive (green), negative (red), positive and negative (brown), or undefined (black) are shown.

**Figure S3: *tra1_Q3_* cells display reduced longevity, impaired growth on glycerol and increased sensitivity to oxidative stress in the BY4742 background.**  **(A)** *TRA1* and *tra1_Q3_* cells were grown for the indicated time in standard synthetic complete medium and spotted on agar plates. *TRA1* and *tra1_Q3_* cells at day 1 were also spotted on agar plates containing glycerol as the carbon source. **(C)** *tra1_Q3_* cells are sensitive to diamide. *TRA1* and *tra1_Q3_* cells were spotted onto agar plates without (untreated) or with 1 mM diamide.
