# Supplementary material for: Tra1 controls the transcriptional landscape of the aging cell
Source: G3 (Bethesda). 2022 Oct 31;13(1):jkac287. doi: 10.1093/g3journal/jkac287 (PMC9836359; doi:10.1093/g3journal/jkac287)
Supplement: jkac287_Supplementary_Data [file jkac287_supplementary_data.zip › Suppl/Table_S1_G3-2022-403832.docx]

**Table S1**

| Strain | Genotype | plasmid | References |
| --- | --- | --- | --- |
| YPL611 | *W303a derivative*  *MATa ade2-1 can1-100 trp1-1 leu2-3 his3-11 ura3-1*  *URA3-Flag^5^-TRA1-HIS3* | YCplac111-  *DED1pr-YHR100* | This study |
| YPL612 | *W303a derivative*  *MATa ade2-1 can1-100 trp1-1 leu2-3 his3-11 ura3-1*  *URA3-Flag^5^-TRA1-HIS3* | YCplac111-  *DED1pr-YHR100* | This study |
| CY4353 | *MATα his3Δ0 leu2Δ0*  *ura3Δ0 TRA1-HIS3* | YCplac111-  *DED1pr-YHR100* | [(Hoke *et al.* 2008)](https://paperpile.com/c/MAGTIx/UFg5) |
| CY6582 | *MATα his3Δ0 leu2Δ0*  *ura3Δ0 tra1_Q3_-HIS3* | YCplac111-  *DED1pr-YHR100* | [(Berg *et al.* 2018)](https://paperpile.com/c/MAGTIx/SE89) |
