# Supplementary figures and images for: Tra1 controls the transcriptional landscape of the aging cell
Source: G3 (Bethesda). 2022 Oct 31;13(1):jkac287. doi: 10.1093/g3journal/jkac287 (PMC9836359; doi:10.1093/g3journal/jkac287)

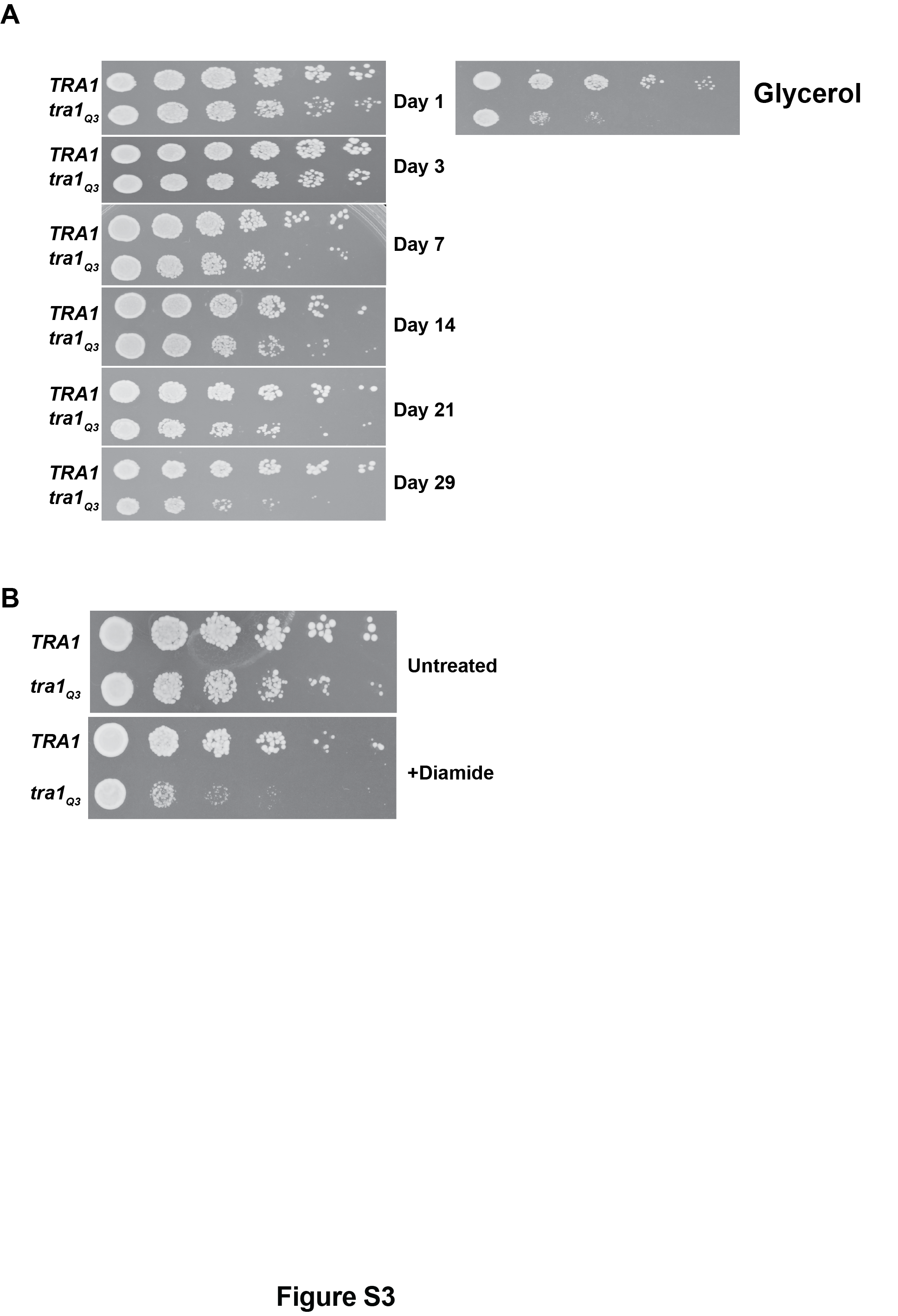

Supplement: jkac287_Supplementary_Data [file jkac287_supplementary_data.zip › Suppl/Figure_S3_G3-2022-403832.png]

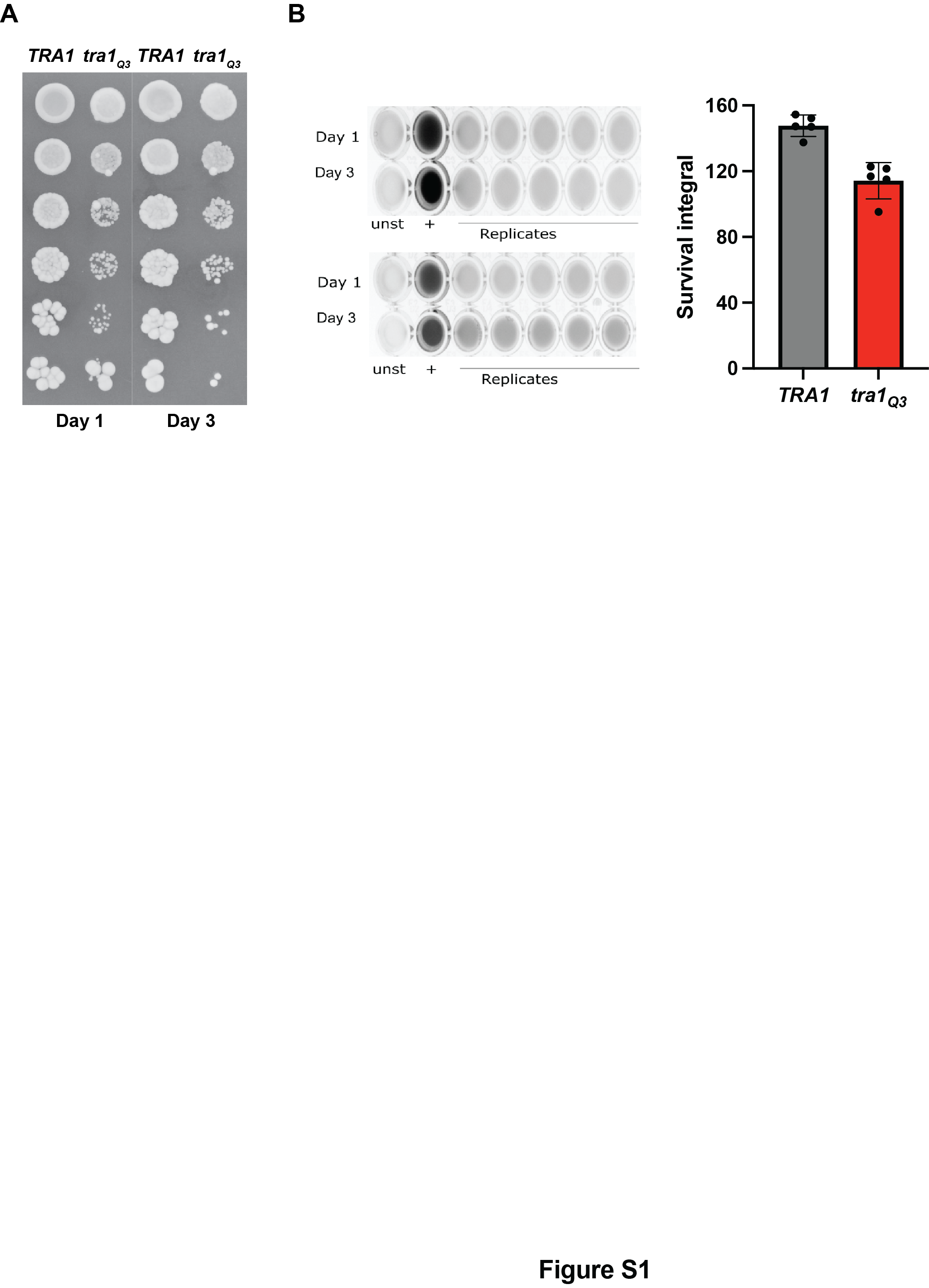

Supplement: jkac287_Supplementary_Data [file jkac287_supplementary_data.zip › Suppl/Figure_S1_G3-2022-403832.png]

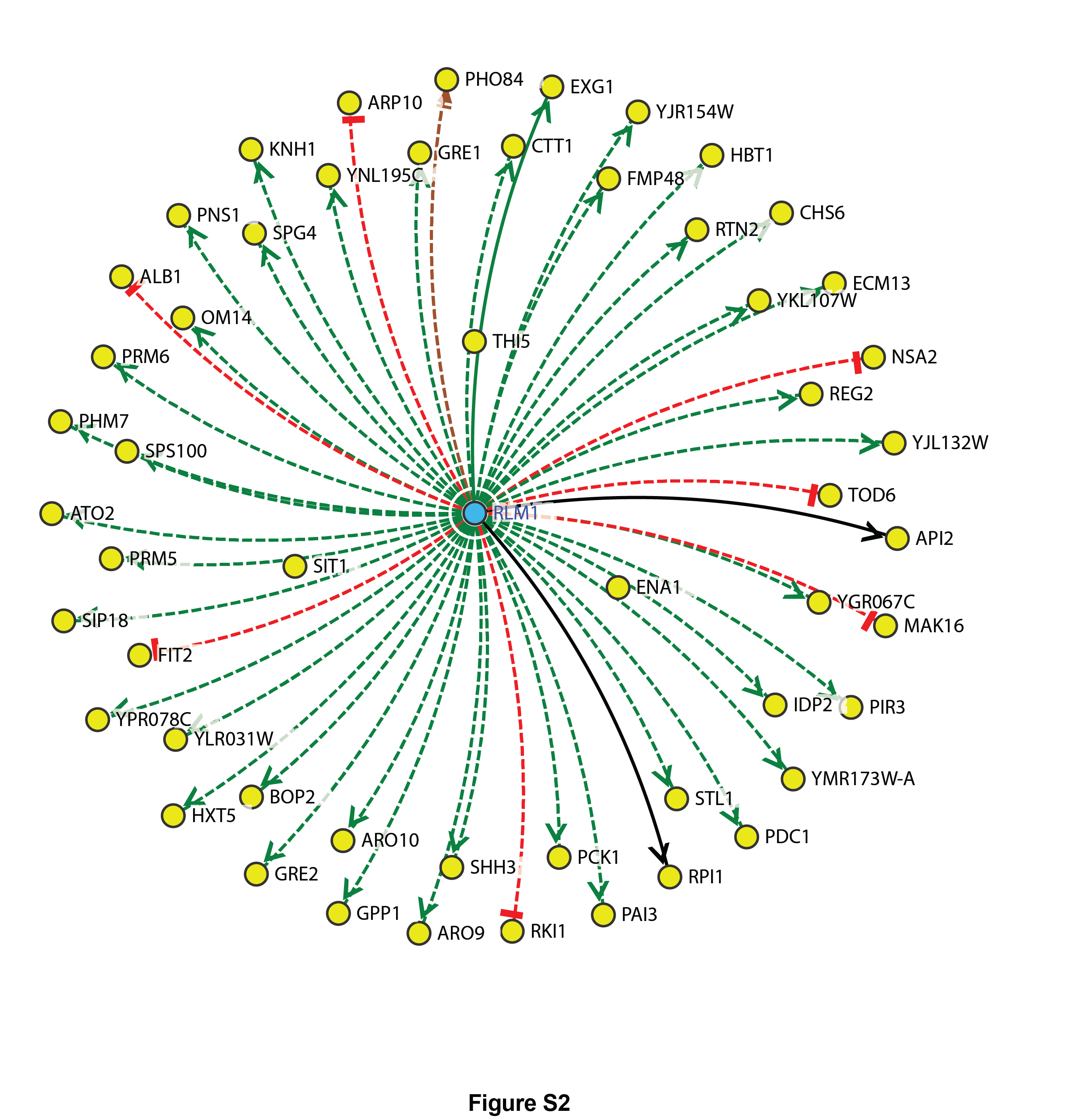

Supplement: jkac287_Supplementary_Data [file jkac287_supplementary_data.zip › Suppl/Figure_S2_G3-2022-403832.png]
